# Microcystins and *Microcystis aeruginosa* PCC7806 extracts modulate steroidogenesis differentially in the human H295R adrenal model

**DOI:** 10.1371/journal.pone.0244000

**Published:** 2020-12-15

**Authors:** Vittoria Mallia, Steven Verhaegen, Bjarne Styrishave, Gunnar Sundstøl Eriksen, Malene Louise Johannsen, Erik Ropstad, Silvio Uhlig

**Affiliations:** 1 Toxinology Research Group, Norwegian Veterinary Institute, Oslo, Norway; 2 Department of Chemistry, University of Oslo, Oslo, Norway; 3 Faculty of Veterinary Medicine, Department of Production Animal Clinical Sciences, Norwegian University of Life Sciences, Oslo, Norway; 4 Faculty of Health and Medical Sciences, Toxicology and Drug Metabolism Group, Department of Pharmacy, University of Copenhagen, Copenhagen, Denmark; Xiangtan University, CHINA

## Abstract

The aim of this study was to investigate the potential interference of cyanobacterial metabolites, in particular microcystins (MCs), with steroid hormone biosynthesis. Steroid hormones control many fundamental processes in an organism, thus alteration of their tissue concentrations may affect normal homeostasis. We used liquid chromatography–tandem mass spectrometry (LC–MS/MS) to investigate the modulation of 14 hormones involved in the adrenal steroid biosynthesis pathway using forskolin-treated H295R cells, following exposure with either microcystin-LR (MC-LR) alone, a mixture made up of MC-LR together with eight other MCs and nodularin-R (NOD-R), or extracts from the MC-LR-producing *Microcystis aeruginosa* PCC7806 strain or its MC-deficient mutant PCC7806*mcyB−*. Production of 17-hydroxypregnenolone and dehydroepiandrosterone (DHEA) was increased in the presence of MC-LR in a dose-dependent manner, indicating an inhibitory effect on 3β-hydroxysteroid dehydrogenase (3β-HSD). This effect was not observed following exposure with a MCs/NOD-R mixture, and thus the effect of MC-LR on 3β-HSD appears to be stronger than for other congeners. Exposure to extracts from both *M*. *aeruginosa* PCC7806 and *M*. *aeruginosa* PCC7806*mcyB−* had an opposite effect on 3β-HSD, i.e. concentrations of pregnenolone, 17-hydroxypregnenolone and DHEA were significantly decreased, showing that there are other cyanobacterial metabolites that outcompete the effect of MC-LR, and possibly result instead in net-induction. Another finding was a possible concentration-dependent inhibition of CYP21A2 or CYP11β1, which catalyse oxidation reactions leading to cortisol and cortisone, by MC-LR and the MCs/NOD-R mixture. However, both *M*. *aeruginosa* PCC7806 and *M*. *aeruginosa* PCC7806*mcyB−* extracts had an opposite effect resulting in a substantial increase in cortisol levels. Our results suggest that MCs can modulate steroidogenesis, but the net effect of the *M*. *aeruginosa* metabolome on steroidogenesis is different from that of pure MC-LR and independent of MC production.

## Introduction

Cyanobacteria, also commonly known as “blue–green algae”, represent an increasing concern for wildlife and human health [1–3]. They are photosynthetic prokaryotic inhabitants of aquatic environments worldwide, mainly in freshwater basins. Under certain conditions (e.g. abundance of nutrients, favorable light and temperature), cyanobacteria may over-accumulate, forming so-called “blooms” [3]. Blooming cyanobacteria may release harmful bioactive metabolites into the water, including potent toxins (cyanotoxins) [1,2,4–7].

MCs are likely the most studied among cyanobacterial toxins [8]. They are a large family of cyclic heptapeptides, sharing a common core structure [8–11]. As a result of variations in their amino acid composition and additional structural modifications, MCs are structurally diverse with at least 279 congeners already reported [9]. Several cyanobacterial genera have the ability to produce MCs, including *Microcystis* spp. [8]. The toxicity of MCs is primarily connected to the inhibition of protein phosphatases PP1 and PP2A [12–14] These are ubiquitously expressed and crucial for regulation of key proteins, which in turn is determinant for key cellular processes [15–17].

MCs are known as hepatotoxins, especially following a well-documented event in 1996, when more than fifty patients died of acute liver failure following hemodialysis with MC-contaminated water in Brazil [18,19]. Literature reports several other adverse effects that MCs can exert, both in humans and wildlife, including reproductive toxicity and endocrine disrupting (ED) activity [9,18,20–24], which is the interference with normal homeostasis of the endocrine (hormonal) system [25].

It has been reported that extracts and exudates from cyanobacterial blooms have ED activity, in particular estrogenic activity, through activation of the estrogen receptor [26,27]. However, it is not entirely clear which of the many compounds present in cyanobacterial blooms exert estrogen activity and what role MCs might have. Furthermore, there are several non-receptor-mediated mechanisms that may alter endocrine functions [28,29], including modulation of hormone production at the glandular level [30].

The adrenal cortex, which is the part of the adrenal gland where steroid hormones are produced, has been identified as a major target organ affected by endocrine disruptors (EDs) [31]. Steroid hormones regulate several functions including growth and development, metabolism and reproduction. The overall process of steroid hormone production is called steroidogenesis. It includes several steps and utilizes cholesterol as the common precursor. Cholesterol is transported to the inner mitochondrial membrane by steroidogenic acute regulatory protein (StAR) [32]. Following this rate-limiting step, several key enzymes modify cholesterol into other steroid hormones [33–35].

Most studies into the toxicology of cyanobacteria, including those addressing their ED activity, have focused on microcystin-LR (MC-LR) [13,20–22,24,36], which is considered one of the most prevalent and toxic MC congeners [8,9]. Thus, it is the only congener for which the World Health Organization (WHO) has recommended a limit for its concentration in drinking water (1 μg/L) [37]. A recent study by Wang and co-authors showed that persistent MC-LR exposure in adult male zebrafish increased serum cortisol levels by modulating the expression of hypothalamic-pituitary-interrenal (HPI)-axis genes [38]. In an earlier report, Hou et al. [39] indicated that MC-LR could elicit non-dose-dependent estrogenic effects interfering with steroidogenic gene expression. The latter study was performed using the H295R steroidogenesis assay. The adrenocortical human cell line H295R is an *in vitro* model that conserves physiological characteristics of the steroidogenesis. Thus, these cells have the unique property of expressing genes that encode for all the key steroidogenesis enzymes [28,40–44]. The H295R assay has been optimized and validated by the Organization for Economic Co-operation and Development (OECD) to detect substances that affect production of 17β-estradiol and testosterone [45].

In this study, we employed the H295R model, but focused on the modulation of individual steroids rather than gene expression as in Hou et al. [39]. We investigated the production of 14 hormones of the steroidogenesis pathways after exposing the cells to MC-LR in a concentration range that overlapped with that reported by Hou and co-workers [39], and quantified the hormones by liquid chromatography–tandem mass spectrometry (LC–MS/MS). We also investigated exposures to MC-LR in a mixture together with eight other MC congeners and the closely related pentapeptide, nodularin-R (NOD-R) [8,14,46]. Furthermore, we included exposures to extracts from the *M*. *aeruginosa* PCC7806 strain and its MC-deficient mutant PCC7806*mcyB−* [47]. PCC7806 has been widely employed in other research on cyanobacteria, including in studies on their ED activity [7,23,48–53]. It produces MC-LR as the major MC congener, together with lesser amounts of [D-Asp^3^]MC-LR, including a range of known other bioactive metabolites such as cyanopeptolins, aerucyclamides and aeruginosins, as well as unknown compounds [50–52]. In culture, the *mcyB−* mutant grows similarly to the wild-type, but produces higher concentrations of some compounds other than MCs in a kind of compensatory mechanism [54].

The objective of performing these different exposures was to get a clearer picture of the involvement of MCs in general, and MC-LR in particular, on steroidogenesis, and to clarify whether there are other cyanobacterial metabolites that may modulate hormone concentrations in the H295R model. Thus, the exposure of the cells with pure MC-LR, MC-LR in a mixture with other congeners, an extract from a MC-LR producing cyanobacterial culture or its MC-deficient mutant, was expected to give detailed insight into the role of MC-LR, or other cyanobacterial metabolites, on steroidogenesis.

## Materials and methods

### Chemicals and reagents

Methanol (MeOH, gradient quality) and acetonitrile for extraction of cyanobacteria and protein precipitation, respectively, were from Romil (Cambridge, UK). Water for other purposes than LC–MS was purified and deionized using an ELGA Purelab Maxima system (Vivendi Water Systems, High Wycombe, UK). For H295R cell exposure, the following MC standards (≥ 95% purity) were from Enzo Life Sciences (Enzo Biochem, Inc., Farmingdale, NY, USA): Hepatotox Set 1 (containing MC-LR, MC-RR, MC-LY, MC-YR, MC-LW, MC-LF, MC-LA, and NOD-R), [D-Asp^3^,Dhb^7^]MC-RR (wrongly supplied as [D-Asp^3^]MC-RR) [55]) and [D-Asp^3^]MC-LR. Individual stock solutions of 12.5 μg/mL (MC-LY, MC-LW, MC-LF, MC-LA, [D-Asp^3^,Dhb^7^]MC-RR, [D-Asp^3^]MC-LR, 25 μg/mL (MC-RR), and 50 μg/mL (MC-LR, NOD-R), were prepared in 50% MeOH. From those solutions, a pooled working stock solution containing 1 μg/mL of each compound was prepared in 100% MeOH and diluted to the concentrations needed for preparation of samples for cell exposures (S1 Scheme). Dulbecco's Modified Eagle Medium/Nutrient Mixture F-12 (DMEM/F-12, Gibco, Thermo Fisher Scientific, Waltham, MA, USA) and trypsin containing 0.25% EDTA (Gibco), were from Fisher Scientific (Trondheim, Norway). Forskolin (from *Coleus forskohlii*, ≥ 98% (HPLC), powder) and charcoal-stripped foetal bovine serum (FBS) were from Sigma-Aldrich (Merck KGaA, Darmstadt, Germany). Alamar Blue solution was purchased from Thermo Fischer Scientific (Waltham, MA, USA). Insulin-transferrin-selenium (ITS 500x) was purchased from BioNordika (Oslo, Norway). For LC–MS analyses, HPLC grade MeOH, acetonitrile and water was from VWR Chemicals (Søborg, Denmark), while formic acid (98–100%) was from Merck KGaA and of EMSURE® quality. Steroid standards were from Sigma-Aldrich and of >96% purity. Deuterated internal standards were either from Toronto Research Chemicals (North York, ON, Canada), CDN isotopes (Pointe-Claire, QC, Canada) or Sigma-Aldrich.

### Cyanobacterial material

#### Cultivation of cyanobacteria

Axenic *M*. *aeruginosa* PCC7806 and its MC-deficient mutant PCC7806*mcyB−* were from the Pasteur Institute (Paris, France). They were cultivated in sterile Z8 medium [56] in 100 mL sterilized glass Erlenmeyer flasks in an incubator (IPP110plus, Memmert GmbH + Co.KG, Schwabach, Germany) at 18°C with a 14/10 h light/dark photoperiod, using 1% of maximum light intensity.

#### Cyanobacterial extract preparation

Cultures of *M*. *aeruginosa* PCC7806 and *M*. *aeruginosa* PCC7806*mcyB−* were extracted when they were visually dense. For extract preparation, 5 mL of each culture was transferred to a glass tube and stored at −20°C overnight, then allowed to thaw at room temperature, and 5 mL of MeOH was added. The tube was subsequently vortex-mixed for 20 s, sonicated for 5 min and centrifuged for 10 min at 1,000 rcf. The supernatant was transferred to glass containers and stored at −20°C until use.

### H295R cell culture and exposure

#### Culture conditions

The H295R cell line was from American Type Culture Collection (ATCC). Cells were cultured in 75 cm^2^ flasks in DMEM/F-12, containing HEPES buffer, L-glutamine and phenol red. Additional supplements were added: FBS at 5% and ITS 500x at 0.2%. H295R cells were incubated at 37°C, in 5% CO_2_ under a humidified atmosphere (Air-Jacketed, DH Autoflow Automatic CO_2_ Incubator; NuAire, Fernbrook, MN, USA), changing the medium every 2–3 days, depending on the density of cells. Cells were passaged when approximately 80% confluent by trypsination using 0.25% trypsin-EDTA. The cells were used for experiments between passages 9 and 10.

#### Preparation of test compounds and cyanobacterial extracts

Concentrated stock solutions of individual test compounds were prepared in MeOH. In the assay dilutions, the concentration of MeOH was kept constant at 0.5% to avoid solvent-related cytotoxicity. MC-LR was tested at concentrations of 1, 5, 100, 500, and 1000 ng/mL. The mixture of standard MCs and NOD-R was tested at assay concentrations of 1, 5 and 100 ng/mL (of each toxin, resulting in total toxin concentrations of 10, 50 and 1000 ng/mL, respectively). The MC concentration in the *M*. *aeruginosa* PCC7806 extract was established using liquid chromatography coupled to high-resolution mass spectrometry (LC–HRMS) and adjusted so the total in-assay MC concentration (MC-LR + [D-Asp^3^]MC-LR]) (in 50% MeOH) was approximately 5 and 500 ng/mL (S1 Scheme). The instrumental method used for quantification of MCs against external calibration curves using standards in 50% MeOH was according to method A described by Mallia et al. [57]. The PCC7806*mcyB−* strain was also tested at two different concentrations. These concentrations were obtained by adjusting the overall signal/noise of metabolites observed in full-scan LC–HRMS chromatograms to the same order of magnitude as that observed for the *M*. *aeruginosa* PCC7806. Cyanobacterial growth medium Z8 [56] was extracted and concentrated using the same protocol as was used for the cyanobacterial cultures, and tested as a separate control sample. More details about samples and exposures can be found in the Supporting Information (S1 Scheme).

#### H295R steroidogenesis assay and exposure

H295R cells were seeded at a concentration of 3 × 10^5^ cells/mL, 1 mL/well in white walled, clear and flat-bottomed 24-well plates (Sigma-Aldrich; Merck), and incubated at 37°C, 5% CO_2_ in a humidified atmosphere. After 24h, cells were visually inspected under a light microscope to ensure there were no unwanted inter and/or intra variations in cell monolayer morphology. Then, the seeding medium was replaced by fresh medium, which contained forskolin (1.5 μM) to start stimulating cells directly before exposure, in all wells except for medium and solvent controls. Cells were then exposed to test compounds and cyanobacterial extracts, as well as Z8 cyanobacterial growth medium [56] (see below). Each experimental plate included medium control (cell growth medium), solvent control (cell growth medium, containing 0.5% MeOH) and positive control (cell growth medium, containing 0.5% MeOH and 1.5 μM forskolin) in triplicate (S1 Scheme). Medium controls and solvent controls were used to verify cell performance during the experiments, ensuring that the cells were not affected by unwanted external effects or technical bias. Positive controls were used as a reference for evaluating how test compounds and extracts impacted hormone production. After 48 h of exposure, the medium from each well (~1 mL) was collected in 2-mL Eppendorf tubes and stored at −80°C until further processing.

#### Cell viability

Potential cytotoxicity was evaluated using the Alamar Blue^TM^ assay (Invitrogen). After 48 h of exposure, the medium was removed from the wells and replaced by 1 mL of fresh medium containing 10% Alamar Blue assay solution. Plates were incubated for 3 h at 37°C, in 5% CO_2_ in a humidified atmosphere, and then 100 μL from each well was transferred to a 96-well plate (Thermo Fisher Scientific) for fluorescence reading using a Spectramax i3x plate reader (Molecular Devices, San Jose, Ca, USA) [58]. Cells with lower viability than 80% compared to solvent control were discarded.

#### Steroid extraction of H295R cell culture medium

Protein precipitation was performed according to Weisser et al. [59] before chemical analysis by LC–MS/MS. Internal standard (IS) solution (50 μL, 0.1 μg/mL per compound) was added to each Eppendorf tube containing the following deuterated steroid analogues in MeOH: d_7_-androstenedione, d_4_-estrone, d_5_-17β-estradiol, d_8_-corticosterone, d_8_-11-deoxycorticosterone, d_9_-progesterone, d_3_-testosterone, d_4_-cortisol, d_5_-11-deoxycortisol and d_6_-dehydroepindrosterone. Thereafter, 900 μL of ice-cold acetonitrile was added, and the tubes vortex-mixed for 20 s. The tubes were stored at −20°C for at least 10 min to allow complete precipitation and then centrifuged at 24532 rcf and 4°C for 20 min. Supernatants were transferred to glass tubes and evaporated at 60°C under a gentle stream of nitrogen to a volume of about 1 mL. For a second protein precipitation, 900 μL of ice-cold MeOH was added and the tubes vortex-mixed for 20 s. Again, tubes were stored at −20°C for at least 10 min, to allow complete precipitation and then centrifuged at 24532 rcf and 4°C for 15 min. Supernatants from each tube (approximately 1.9 mL) were transferred to 2 mL chromatography vials and evaporated at 60°C to approximately 0.7 mL under a gentle stream of nitrogen. Finally, vials were filled up with purified water, to a final volume of 1 mL and stored at −20°C until hormone analyses.

### LC–MS/MS steroid analyses

**Fig 1:**
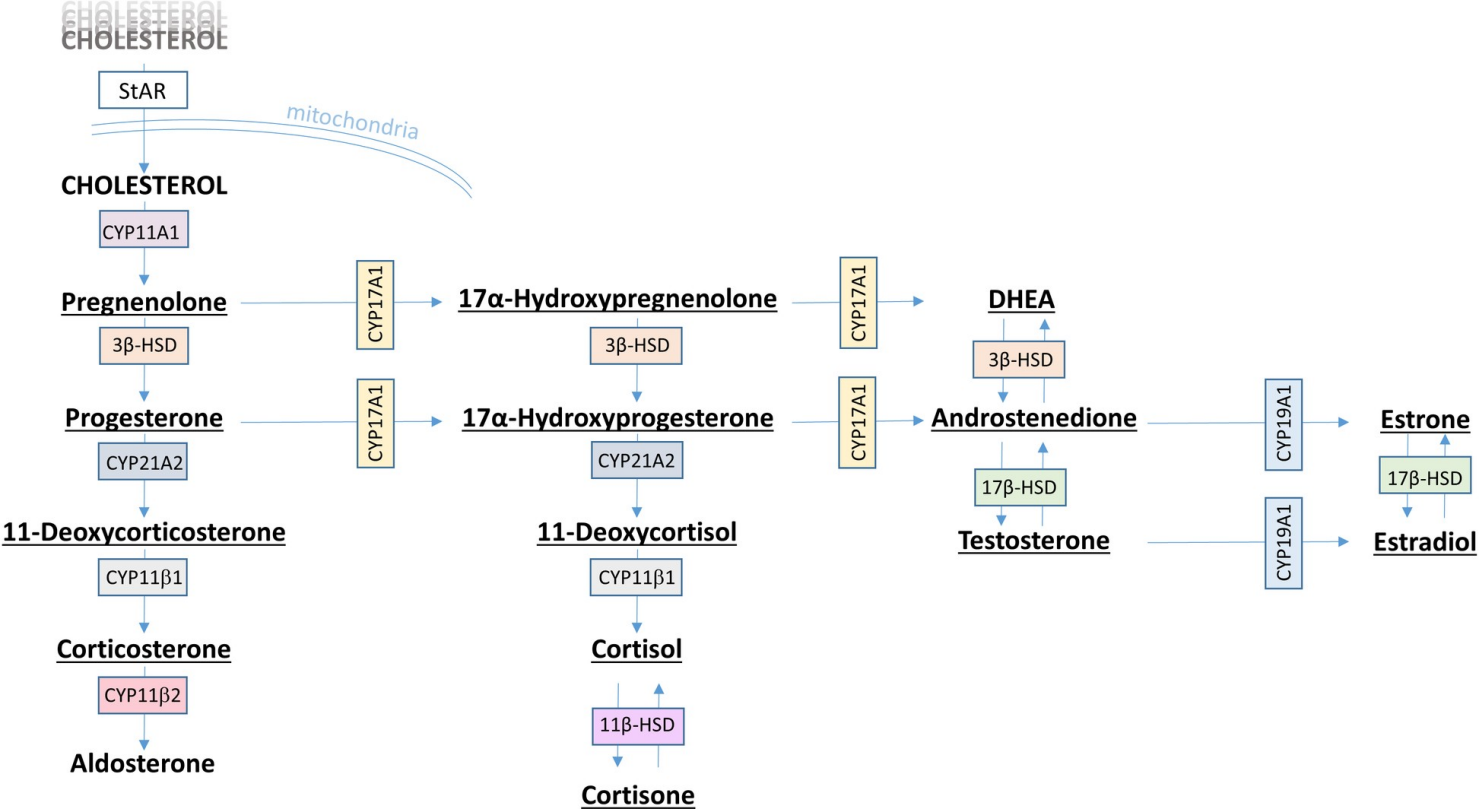
Steroidogenesis pathway. Steroidogenesis pathway, including hormones and enzymes involved [32]. The 14 hormones analysed in this study are underlined.

The samples collected from the H295R cell assay were analyzed for 14 steroid hormones involved in the steroidogenesis (Fig 1) using LC–MS/MS according to earlier work [59].

Briefly, a combination of a binary 1290 Agilent Infinity system and a binary 1100 Agilent HPLC pump was used for on-line clean-up and chromatographic separation of steroids. Initially, 100 μL of each sample was injected onto an octadecylsilane column (3.9 x 20 mm, 10 μm particles) for purification. Steroids were retained while impurities were washed directly into waste. For chromatographic separation, steroids were loaded on to an octadecylsilane analytical column with guard column thermostatted at 40°C by gradually increasing the proportion of MeOH in acidic water (0.1% (v/v) formic acid in water) utilizing a flow rate of 0.3 mL/min. For the detection and identification of steroids an AB Sciex 4500 QTRAP tandem quadrupole mass spectrometer was used (AB Sciex LLC, Toronto, Canada). The analytes were ionized by atmospheric pressure chemical ionization (APCI) in the positive ionization mode, and the mass spectrometer operated in the multiple reaction monitoring (MRM) mode. Data collection and processing were performed using the Analyst 1.6.2 software package (AB Sciex).

The analytical method had been validated according to the ICH (2005) guideline [60] on bioanalytical method validation. A standard curve for each steroid was plotted according to Weisser et al. [59]. To evaluate and verify the performance of the instrument, blanks and standards were run for every 6 and 12 samples, respectively. The lower limits of detection for the method were determined for the current batch of samples and were in the range 0.048 ng/mL for testosterone to 0.228 ng/mL for DHEA (Table 1). More comprehensive information on general method performance may be found in Weisser et al. [59].

**Table 1:** Limits of detection (LOD) and limits of quantification (LOQ) for individual steroid hormones using LC-MS/MS.

Steroid	LOD (ng/mL)	LOQ (ng/mL)
11-deoxycortisol	0.072	0.218
11-deoxycorticosterone	0.103	0.311
17-hydroxypregnenolone	0.211	0.639
17-hydroxyprogesterone	0.161	0.487
androstenedione	0.090	0.273
cortisol	0.049	0.148
cortisone	0.061	0.185
corticosterone	0.053	0.159
DHEA	0.228	0.691
estrone	0.084	0.254
17β-estradiol	0.137	0.414
pregnenolone	0.134	0.406
progesterone	0.123	0.373
testosterone	0.048	0.144

#### Data processing and statistical analysis

The obtained chromatographic peak areas were integrated using MultiQuant 3.0 Software (AB Sciex). The chromatograms from all samples were inspected and, if necessary, integrated manually. The fold-change was calculated for individual steroid concentrations relative to the positive (1.5 μM forskolin) control, and the results plotted in Sigma Plot v. 14 (Systat Software Inc., San Jose, CA, USA).

#### Univariate data analyses

Hormone concentrations for all exposures (Supporting information) were normalized prior to statistical analyses by calculating the difference between individual hormone concentrations and the mean hormone concentrations for the positive (1.5 μM forskolin) control using Microsoft Excel 2016. Univariate data analyses were performed using JMP 14.0 (SAS Institute Inc., Cary, NC, USA). The majority of the data were not normally or log-normally distributed, and we thus used a two-tailed Wilcoxon signed-rank test to assess whether differences in hormone concentrations deviated from zero. A P-value of <0.05 was considered significant.

#### Multivariate data analyses

Missing concentrations for 11-deoxycorticosterone (Supporting information) from one exposure experiment were assumed by taking the mean of the six concentration readings from the two other experiments. The data were log-transformed in order to reduce noise and impact of high variance, and pareto-scaled by dividing each variable by the square root of its standard deviation using Umetrics SIMCA 15.0 (Sartorius Stedim Data Analytics AB, Umeå, Sweden) prior to multivariate analyses using unsupervised and supervised models. Principal component analysis (PCA) was performed for an initial exploration of the data with the main purpose of detecting patterns and potential outliers. Principal component analysis was also performed using the original non-normalized data in order to assess the homogeneity of medium and solvent controls. Orthogonal partial least-squares discriminant analysis (OPLS-DA) was carried out to identify features (i.e. steroids) that discriminated between exposures and the positive (1.5 μM forskolin) control. The discriminant analysis included a default seven-round cross-validation. Furthermore, cross-validation ANOVA (CV-ANOVA) was performed to assess the reliability of the models. Steroid hormones that contributed to class separation in valid OPLS-DA models were inferred from the model S-plots and the predictive variable importance in the projection (VIP) values. Models were rejected if the *P*-value from CV-ANOVA was higher than 0.05. Furthermore, models were regarded as robust when *R*^*2*^*Y* and *Q*^*2*^*X* were higher than 50% and 40%, respectively [61,62].

## Results

Of the 14 analysed hormones in the H295R cell culture medium, we were able to acquire quantitative data for 13 steroids. Corticosterone was not detected above the lower limit of detection (0.023 ng/mL) in any of the samples.

### Univariate analyses

**Fig 2:**
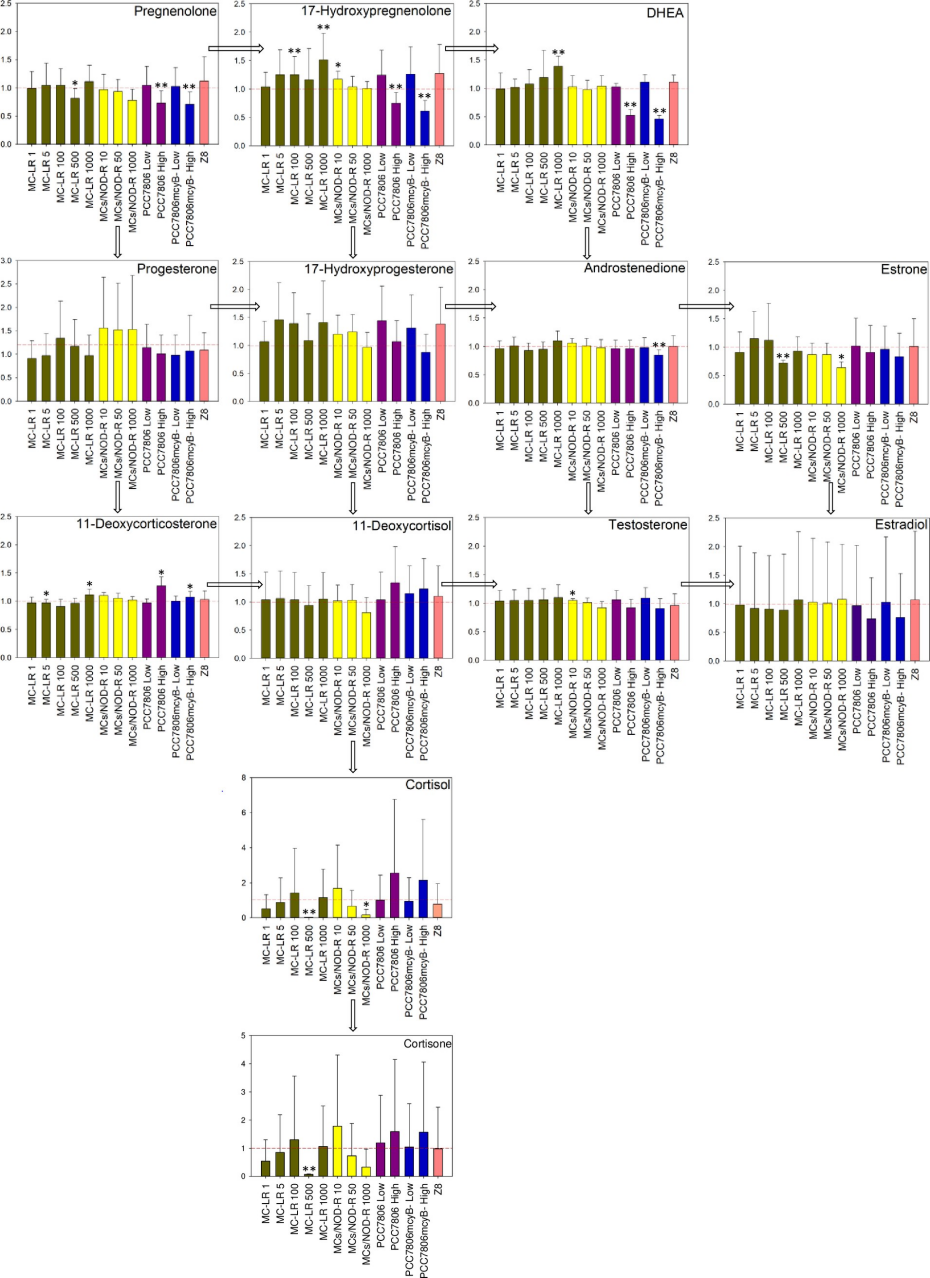
Modulation of individual steroids including results from univariate statistical analysis. Modulation of hormone production in H295R cells following exposure to different concentrations of MC-LR, a mixture of nine MCs and NOD-R (MCs/NOD-R), the MC-producing strain *M*. *aeruginosa* PCC7806 or its MC-deficient PCC7806*mcyB−* mutant, or the cyanobacterial growth medium Z8 [56] for 48 h. The in-assay MC concentration (MC-LR + [D-Asp^3^]MC-LR) in the *M*. *aeruginosa* PCC7806 exposures were adjusted to approximately 5 ng/mL (“low”) and 500 ng/mL (“high”). MC-deficient PCC7806*mcyB−* exposures were adjusted so the LC–HRMS response of metabolites other than MCs were in the same order of magnitude as for corresponding *M*. *aeruginosa* PCC7806 exposures. Data are presented as fold change relative to the positive (1.5 μM forskolin) control (which is represented by the dashed red line). The number of independent repeats was 3. Error bars represent the standard deviation, and asterisks show significant differences from the positive control at a 5% (*) or 1% (**) level of significance based on the Wilcoxon signed-rank test.

Exposure to the high-concentration extracts of *M*. *aeruginosa* PCC7806 and *M*. *aeruginosa* PCC7806*mcyB−*, and to higher concentrations of MC-LR or MCs/NOD-R, were associated with significant changes in several steroids (Fig 2). Steroid hormone profiles following exposure with both high-concentration *M*. *aeruginosa* PCC7806 and *M*. *aeruginosa* PCC7806*mcyB−* extracts showed a similar pattern. In both cases, dehydroepiandrosterone (DHEA), 17-hydroxypregnenolone and pregnenolone were detected with significantly lower concentrations relative to the positive forskolin control (Fig 2). In addition, androstenedione levels were significantly lower following exposure to *M*. *aeruginosa* PCC7806*mcyB−* extract (fold change 0.85) (Fig 2). In the high-concentration exposures with both *M*. *aeruginosa* strains, concentrations of 11-deoxycorticosterone were significantly higher relative to the positive forskolin control (fold change 1.27 and 1.07 for PCC7806 and PCC7806*mcyB−*, respectively). The steroid 11- deoxycorticosterone was also significantly upregulated following exposure to 1000 ng/mL MC-LR (fold change 1.11) (Fig 2). However, in contrast to the *M*. *aeruginosa* PCC7806 exposures, production of DHEA and 17-hydroxypregnenolone was significantly higher following exposure with 1000 ng/mL MC-LR (also 100 ng/mL in case of 17-hydroxypregnenolone), compared to the positive control (fold change 1.39 and 1.51, respectively). There was no such concentration-dependent increase in DHEA and 17-hydroxypregnenolone when H295R cells were exposed to MCs/NOD-R (Fig 2).

A second effect of our exposures was on the pathway that leads from 17-hydroxypregnenolone and via 17-hydroxyprogesterone and 11-deoxycortisol to cortisol and cortisone (Fig 2). This effect was strongest for the MCs/NOD-R mixture and culminated in significantly reduced cortisol (fold change 0.16 relative to positive control) at 1000 ng/mL total toxin concentration (including MC-LR at 100 ng/mL) (Fig 2). The same effect was observed for the 500 ng/mL exposure with MC-LR that significantly (p<0.01) reduced cortisol and cortisone by 97% and 93%, respectively (Fig 2). Again, both *M*. *aeruginosa* PCC7806 extracts, i.e. its wild-type and the MC-deficient *mcyB−* mutant, showed an opposite effect. Thus, the higher-concentration exposures with *M*. *aeruginosa* PCC7806 and *M*. *aeruginosa* PCC7806*mcyB−* extracts substantially increased cortisol with fold changes of 2.55 and 2.15, respectively (Fig 2). This effect was not statistically significant due to high variability of cortisol concentrations between experiments.

### Multivariate analyses

**Fig 3:**
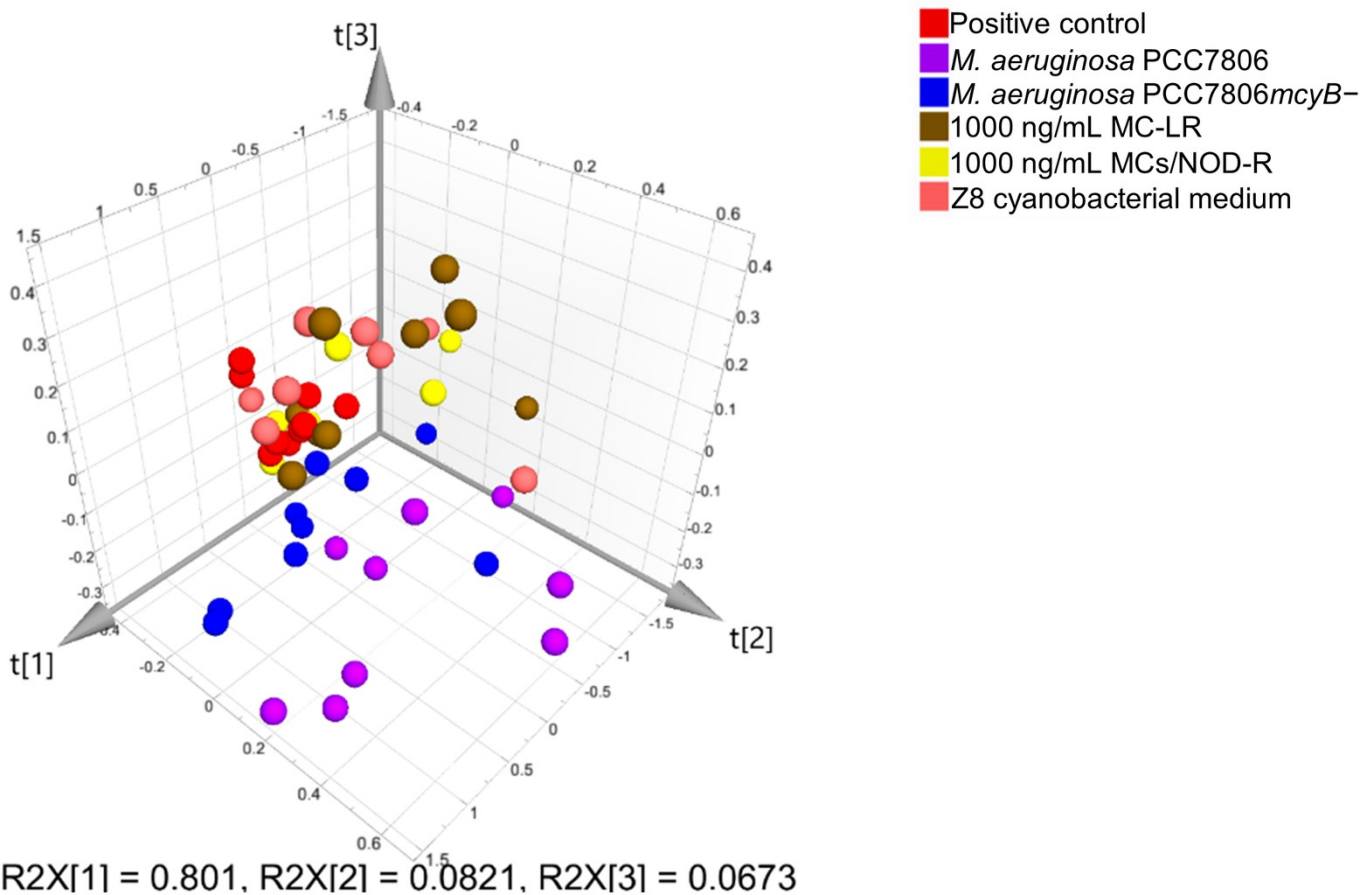
Principal component analysis scores plot including data from high-concentration exposures. 3-D scores plot from unsupervised principal component analysis based on pareto-scaled and positive-control-subtracted hormone concentrations, from high-exposure groups only. The first three components explained 95% of the total variation. The plot shows a larger dispersion of the data from *M*. *aeruginosa* PCC7806 exposures, but individual observations from these exposures also clustered further away from the positive control and microcystin exposures.

Principal component analysis (PCA) was carried out in order to visualize data structure within and between exposure experiments. Solvent and medium controls formed a separate and relatively homogeneous cluster across experiments showing the absence of technical bias (S1 Fig). As hormone levels within the same exposure groups varied considerably between experiments (S1 Fig), a final PCA model was limited to the highest exposures only and based on the positive control normalized data (i.e. the identical data set that was used for the univariate analysis). The resulting PCA scores plots show that the first three principal components explained 95% of the variation in the 13 measured hormones (cumulative *Q*^*2*^*X* for the three-component model = 43%) (Fig 3). Only the *M*. *aeruginosa* PCC7806 exposures lead to changes in the hormone profiles that could be visualized in the PCA scores plot (Fig 3). Thus, both the PCC7806 and the MC-deficient PCC7806*mcyB−* mutant clustered separately from all other exposures, but individual observations were also more dispersed. The 1000 ng/mL MC-LR or MCs/NOD-R exposure groups did not form separate clusters and were partially overlapping with the positive control or the Z8 cyanobacterial growth medium (Fig 3).

**Fig 4:**
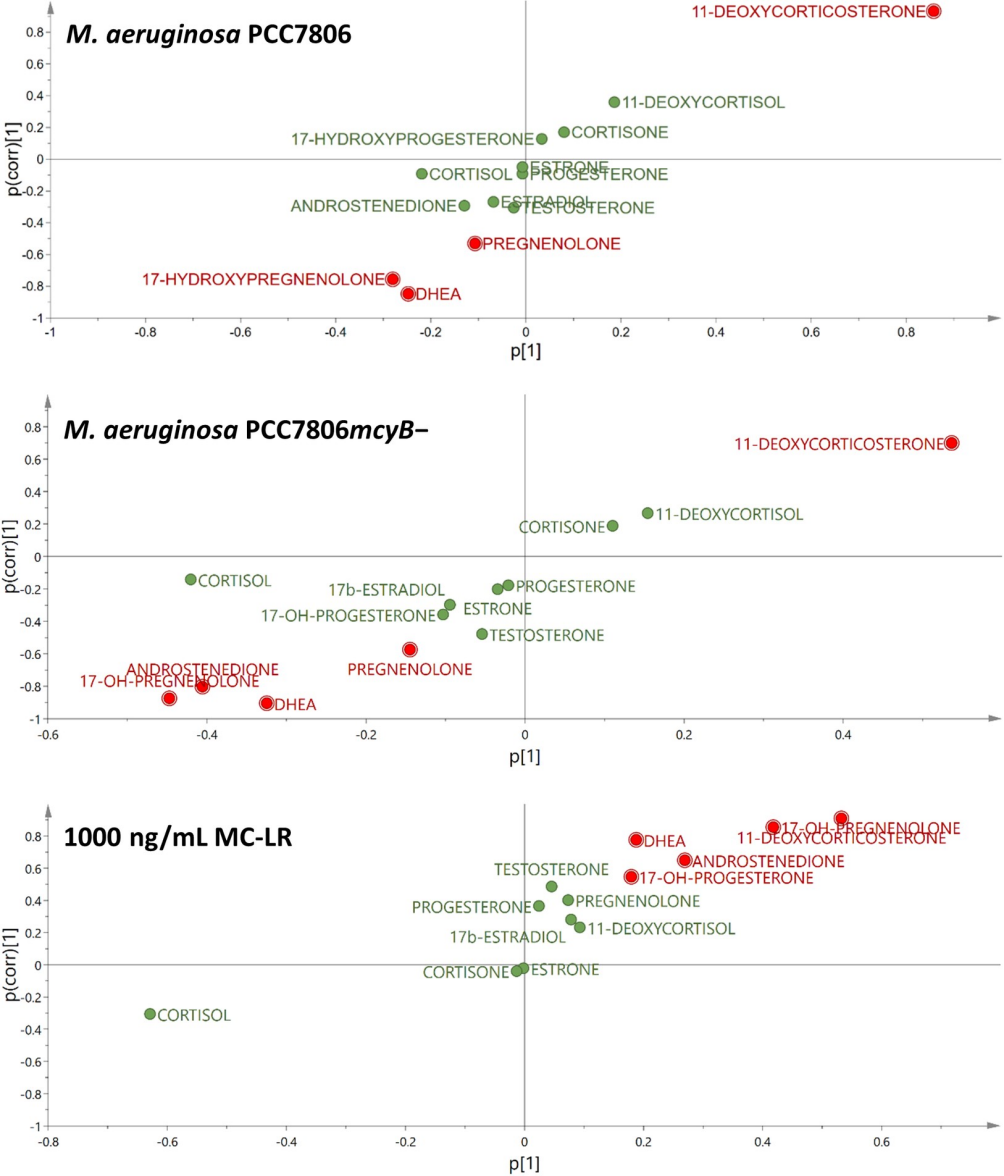
S-plots from valid orthogonal partial least squares discriminant analysis (OPLS-DA) models. S-plots from two-class OPLS-DA models including observations for positive (1.5 μM forskolin) control versus either the wild type of *M*. *aeruginosa* PCC7806 (upper plot), its MC-deficient mutant PCC7806*mcyB−* (middle plot), or 1000 ng/mL MC-LR (lower plot). The plots show that 11-deoxycorticosterone was upregulated for all exposures. Dehydroepiandosterone (DHEA) and 17-hydroxypregnenolone were downregulated in exposures for both *M*. *aeruginosa* PCC7806 strains but upregulated following MC-LR exposure. Related hormones, i.e. androstenedione and pregnenolone followed the same pattern but were not among the main discriminating variables in all three models. Cortisol was not included in the selection of significant variables as it was the major contributing factor to orthogonal variation (oVIP = 3.5).

Subsequently, two-class OPLS discriminant analyses were performed, including the positive (1.5 μM forskolin) control as one of the test groups, with the aim of identifying variables (i.e. steroids) with class-separating power. Three models were robust and met the quality criteria outlined above; these were from discriminant analysis of positive control vs. both of the high-concentration *M*. *aeruginosa* PCC7806 extracts (i.e. wild-type and *mcyB−*) and from positive control vs. 1000 ng/mL MC-LR (Table 2). Significant models included between two and three components and interpreted at least 91% of the variation in *X* (i.e. the hormone levels). The predictive value of the three models was medium to high with cumulative *Q*^*2*^*X* values higher than 52% (Table 2). Hormones that were predictive for the observed class separation were extracted from the OPLS-DA S-plots (Fig 4) [63,64]. The strongest predictive hormone for class separations was 11-deoxycorticosterone (OPLS-DA predictive VIP = 1.9–3.1), which was upregulated in H295R cells following exposure both with 1000 ng/mL MC-LR and the high-concentration *M*. *aeruginosa* PCC7806 extracts (Fig 4). In contrast, several steroid hormones were downregulated following exposure with the high-concentration *M*. *aeruginosa* PCC7806 extracts, but upregulated following exposure with 1000 ng/mL MC-LR (Fig 4 and Table 2). Thus, both 17-hydroxypregnenolone and DHEA were significantly discriminating variables in all three OPLS-DA models, but with opposite sign in the 1000 ng/mL MC-LR exposure relative to exposure with the high-concentration *M*. *aeruginosa* PCC7806 and *M*. *aeruginosa* PCC7806*mcyB−* extracts. Furthermore, both their direct precursor, pregnenolone, and biosynthetic product, androstenedione, were significantly discriminating variables in two out of the three models (Fig 4 and Table 2).

**Table 2:** Characteristics of valid two-class OPLS-DA models based on quantitative hormone concentrations from 13 hormones following H295R cell exposures with either 1000 ng/mL MC-LR, *M*. *aeruginosa* PCC7806 or PCC7806*mcyB−* extracts, compared to the positive control (1.5 μM forskolin).

Classes	R^2^(x) (cum)	R^2^(y) (cum)	Q^2^(x) (cum)	CV-ANOVA *P*-value	Steroids predictive for class difference incl. pVIP^a^
PC vs. MC-LR 1000 ng/mL	0.93	0.58	0.52	0.039	11-deoxycorticosterone (1.9), 17-hydroxypregnenolone (1.5), androstenedione (0.96), DHEA (0.67), 17-hydroxyprogesterone (0.64)
PC vs. PCC7806 (high)	0.97	0.89	0.83	1.1×10^−3^	11-deoxycorticosterone (3.1), 17-hydroxypregnenolone (1.0), DHEA (0.89)
PC vs. PCC7806*mcyB−* (high)	0.91	0.84	0.81	1.3×10^−4^	11-deoxycorticosterone (1.9), 17-hydroxypregnenolone (1.6), androstenedione (1.5), DHEA (1.2), pregnenolone (0.52)

^a^ Selection criteria: predictive Variable Importance for the Projection (pVIP) > 0.5; p[1] >|0.05|; p(corr)[1] >|0.5|.

## Discussion

Exposure of H295R cells with either MC-LR, a mixture of MCs (including MC-LR) and NOD-R or *M*. *aeruginosa* PCC7806 or PCC7806*mcyB−* modulated steroidogenesis in H295R cells in our study (Figs 2–4). A recent study reported the upregulation of testosterone levels in H295R cells following exposure to 10 ng/mL MC-LR, while 17β-estradiol levels were elevated following exposure with 1 and 10 ng/mL MC-LR, but decreased when the cells were exposed with 500–5000 ng/mL [39]. We did not observe an effect on estrogen levels in any of our exposures, which might in part be explained by the high variability in the data for this steroid (Figs 2 and 4). Testosterone was significantly (p<0.05) increased following exposure to 10 ng/mL MCs/NOD-R mixture, but with a rather small fold change (1.05) relative to the positive control (Fig 2). Thus, the detected difference in this case is likely a result of chance (type I error). However, while our cells were treated with 1.5 μM forskolin prior to exposure, the cells were not stimulated in the study by Hou et al. [39]. The use of 1.5 μM forskolin for studying effects on steroidogenesis using H295R cells has previously been suggested as it stimulates hormone synthesis without saturating their production [65]. While MC-LR did not affect 17β-estradiol and testosterone concentrations in forskolin-treated H295R cells, the toxin significantly increased 17-hydroxypregnenolone and DHEA in a concentration-dependent manner, as well as 11-deoxycorticosterone (Fig 2). Furthermore, multivariate statistical modelling in addition showed the significant upregulation of androstenedione and 17-hydroxyprogesterone (Fig 4 and Table 2). The upregulation effect was most obvious (in terms of fold change) for 17-hydroxypregnenolone and DHEA, which both are substrates for 3β-hydroxysteroid dehydrogenase (3β-HSD) [32]. Thus, MC-LR appeared to inhibit 3β-HSD in a dose-dependent manner (Fig 2). This was most clear for DHEA, probably because this hormone has relatively low 3β-HSD affinity and V_max_ conversion rates, allowing for stronger interference by MC-LR [66]. Androstenedione is a substrate of 17β-HSD, and its upregulation could in addition indicate an effect on this enzyme, though much weaker than the effect on 3β-HSD. There was no such concentration-dependent effect for the MCs/NOD-R mixture indicating that the effect of MC-LR on 3β-HSD is stronger than for the other congeners in the tested mixture. The observed significant decrease in estrone levels following exposure with 500 ng/mL MCs/NOD-R mixture or 1000 ng/mL MC-LR supports a possible interaction with 17β-HSD (Fig 2). An effect of MC-LR on the gene expression of 17β -HSD has also been observed in male *Rana nigromaculata* frogs [67]. The same study reports in addition decreased testosterone and increased estradiol levels following long-term exposure (14 days) with 1 μg/L and 0.1 μg/L MC-LR, respectively.

The *M*. *aeruginosa* PCC7806 extract also showed a concentration-dependent effect on 3β-HSD. However, the effect was opposite to that observed for pure MC-LR as both pregnenolone, 17-hydroxypregnenolone and DHEA levels were significantly (p<0.01) downregulated at the higher exposure concentration that contained approximately 500 ng/mL of total MC-LR and [D-Asp^3^]MC-LR (Fig 2). This effect was also evident in the corresponding supervised OPLS-DA model (Fig 4 and Table 2). The *M*. *aeruginosa* PCC7806*mcyB−* extract exhibited the same downregulation effect on pregnenolone, 17-hydroxypregnenolone and DHEA as the wild-type (Figs 2 and 4 and Table 2). This shows that there were other metabolites in the cyanobacterial extracts that had a stronger and opposite effect on 3β-HSD compared to MC-LR. The *M*. *aeruginosa* PCC7806*mcyB−* extract also resulted in significant downregulation of androstenedione indicating that there are cyanobacterial metabolites that also may induce 17β-HSD, though to a lesser extent than 3β-HSD.

A second effect of our exposures was on enzymes that are involved in the pathway that leads from 17-hydroxypregnenolone and via 17-hydroxyprogesterone and 11-deoxycortisol to cortisol and cortisone (Fig 2). This effect was not revealed by the multivariate statistics. Thus, Fig 2 shows a concentration-dependent inhibition effect of both MC-LR and the MCs/NOD-R mixture on CYP21A2 or CYP11β1 that are responsible for conversion of 17-hydroxyprogesterone to 11-deoxycortisol, and of 11-deoxycortisol to cortisol, respectively [32]. Also this effect was reversed by both high-concentration *M*. *aeruginosa* PCC7806 extracts, though not with statistical significance (Fig 2). The reversing effect of the cyanobacterial extracts was quite dramatic. Thus, while exposure with 500 ng/mL MC-LR reduced cortisol to 3% relative to the positive forskolin control, exposure with the high-concentration *M*. *aeruginosa* PCC7806 extract increased it to 255% on average relative to the positive forskolin control.

A statistically significant and concentration-dependent upregulation of 11-deoxycorticosterone was observed for MC-LR and both *M*. *aeruginosa* PCC7806 and *M*. *aeruginosa* PCC7806*mcyB−* extracts, which was evident from both univariate analysis and multivariate modelling. The relative increase was highest for the wild-type *M*. *aeruginosa* PCC7806 (fold change 1.27 relative to positive control) and supports the interaction of both MC-LR, but also other cyanobacterial metabolites with CYP11β1. This result was further supported by non-detectable concentrations of corticosterone, which is the product of the CYP11β1 catalyzed oxidation of 11-deoxycorticosterone.

Our results show that cyanobacterial compounds have ED activity via modulation of steroid biosynthesis. Furthermore, the results indicated an opposite or at least different effect of MCs relative to other cyanobacterial bioactive metabolites. Different effects may reflect different modes of action. MC congeners have a relatively high molecular weight (ca. 1 kDa) and are relatively hydrophilic, thus they may not be regarded as classical EDs. However, they are known to bind PP1 and PP2A, as main mechanisms of toxicity, leading to hyperphosphorylation and interference with key cellular processes. When in complex with PP1 and PP2A, the MC-cycle is twisted, and blocks potential substrates from entering the active site [9,68]. Therefore, it could be hypothesized that a similar mechanism of direct interaction with enzymes involved in steroidogenesis, e.g. HSDs, exist.

The cellular uptake of MCs is mediated by organic anion transporting peptides (OATP) of the OATP1 and OATP2 type [69]. However, H295R cells have only been shown to express OATP3 and OATP4 [70]. In this study, we did not investigate the presence of OATPs in H295R cells, and we did not study the cellular uptake of MCs. Thus, the presented results must be seen in the light of these limitations. An unexpected finding was that also the Z8 cyanobacterial cultivation medium in itself resulted in an obvious response for some steroid hormones, albeit this was not statistically significant (Fig 2). The medium did not contain any chemicals that are known to give endocrine effects [56], and the observed effect was not investigated further.

## Conclusion

Our results show that MCs and other *Microcystis* metabolites may alter steroidogenesis in a dose-dependent manner. MC-LR increased the levels of 17-hydroxypregnenolone and DHEA, while MC-LR alone and in a mixture with eight other MCs and NOD-R resulted in decreased cortisol and cortisone concentrations *in vitro*. Extracts from a MC-producing *Microcystis* strain and its MC-deficient mutant modulated the same hormones but in opposite direction showing that, although MCs in itself can alter steroidogenesis, the extracts contained other bioactive molecules that affected steroidogenesis independently from MC production. One hormone, 11-deoxycorticosterone, was increased following exposure with either MC-LR, extract from MC-producing or MC-deficient *M*. *aeruginosa* indicating that there are bioactivities that are shared by MCs and other cyanobacterial metabolites. As steroidogenesis is complex, including branched pathways and reversible reactions, extrapolation of our findings to exposure of a whole organism with MCs or *Microcystis* is difficult or even impossible.

## Supporting information

S1 Fig Scores plot from principal component analysis of the pareto-scaled and log-transformed H295R hormone data. The purpose of the figure is to show that hormone concentrations of the solvent and medium control samples were homogeneous across experiments thus confirming the absence of technical bias.
(DOCX)

S1 Table Overview over cell viability data. (XLSX)

S2 Table Overview over raw data from hormone analyses. (XLSX)

S1 Scheme Experimental plan for H295R exposure in three 24-well plates. This scheme was repeated in three independent runs (Expt1, Expt2, Expt3). Concentrations reported are concentration of samples in the assay (once diluted in the growth medium).
(DOCX)

## References

[1] Huisman J, Codd GA, Paerl HW, Ibelings BW, Verspagen JMH, Visser PM. Cyanobacterial blooms. Nat Rev Microbiol. 2018;16(8):471–83. 10.1038/s41579-018-0040-1 . 29946124

[2] Chorus I, Bartram J. Toxic Cyanobacteria in Water: a Guide to their Public Health Consequences, Monitoring and Management; E&FN Spon: New York, NY, USA. WHO; 1999.

[3] Merel S, Walker D, Chicana R, Snyder S, Baures E, Thomas O. State of knowledge and concerns on cyanobacterial blooms and cyanotoxins. Environ Int. 2013;59:303–27. Epub 2013/07/31. 10.1016/j.envint.2013.06.013 . 23892224

[4] Buratti FM, Manganelli M, Vichi S, Stefanelli M, Scardala S, Testai E, et al Cyanotoxins: producing organisms, occurrence, toxicity, mechanism of action and human health toxicological risk evaluation. Arch Toxicol. 2017;91(3):1049–130. Epub 2017/01/23. 10.1007/s00204-016-1913-6 . 28110405

[5] Carmichael WW, Azevedo SM, An JS, Molica RJ, Jochimsen EM, Lau S, et al Human fatalities from cyanobacteria: chemical and biological evidence for cyanotoxins. Environ Health Perspect. 2001;109(7):663–8. 10.1289/ehp.01109663 11485863 PMC1240368

[6] Paerl HW, Otten TG. Harmful cyanobacterial blooms: causes, consequences, and controls. Microb Ecol. 2013;65(4):995–1010. Epub 2013/01/15. 10.1007/s00248-012-0159-y . 23314096

[7] Jonas A, Scholz S, Fetter E, Sychrova E, Novakova K, Ortmann J, et al Endocrine, teratogenic and neurotoxic effects of cyanobacteria detected by cellular in vitro and zebrafish embryos assays. Chemosphere. 2015;120:321–7. Epub 2014/08/30. 10.1016/j.chemosphere.2014.07.074 . 25170595

[8] Catherine A, Bernard C, Spoof L, Bruno M. Microcystins and Nodularins. Meriluoto, J, Spoof, L, Cood, GA Handbook of cyanobacterial monitoring and cyanotoxins analysis 2017.

[9] Bouaicha N, Miles CO, Beach DG, Labidi Z, Djabri A, Benayache NY, et al Structural diversity, characterization and toxicology of microcystins. Toxins (Basel). 2019;11(12). Epub 2019/12/11. 10.3390/toxins11120714 . 31817927 PMC6950048

[10] Botes DP, Kruger H, Viljoen CC. Isolation and characterization of four toxins from the blue–green alga, *Microcystis aeruginosa*. Toxicon. 1982;20(6):945–54. 10.1016/0041-0101(82)90097-6 . 6819659

[11] Botes DP, Viljoen CC, Kruger H, Wessels PL, Williams DH. Configuration assignments of the amino acid residues and the presence of N-methyldehydroalanine in toxins from the blue-green alga, Microcystis aeruginosa. Toxicon. 1982;20(6):1037–42. Epub 1982/01/01. 10.1016/0041-0101(82)90105-2 . 6819658

[12] Fontanillo M, Kohn M. Microcystins: synthesis and structure–activity relationship studies toward PP1 and PP2A. Bioorg Med Chem. 2018;26(6):1118–26. 10.1016/j.bmc.2017.08.040 . 28893598

[13] MacKintosh C, Beattie KA, Klumpp S, Cohen P, Codd GA. Cyanobacterial microcystin-LR is a potent and specific inhibitor of protein phosphatases 1 and 2A from both mammals and higher plants. FEBS Lett. 1990;264(2):187–92. 10.1016/0014-5793(90)80245-e . 2162782

[14] Yoshizawa S, Matsushima R, Watanabe MF, Harada K, Ichihara A, Carmichael WW, et al Inhibition of protein phosphatases by microcystins and nodularin associated with hepatotoxicity. J Cancer Res Clin Oncol. 1990;116(6):609–14. 10.1007/BF01637082 . 2174896 PMC12200272

[15] Tournebize R, Andersen SS, Verde F, Doree M, Karsenti E, Hyman AA. Distinct roles of PP1 and PP2A-like phosphatases in control of microtubule dynamics during mitosis. EMBO J. 1997;16(18):5537–49. Epub 1997/10/06. 10.1093/emboj/16.18.5537 9312013 PMC1170186

[16] Garcia A, Cayla X, Guergnon J, Dessauge F, Hospital V, Rebollo MP, et al Serine/threonine protein phosphatases PP1 and PP2A are key players in apoptosis. Biochimie. 2003;85(8):721–6. Epub 2003/10/31. 10.1016/j.biochi.2003.09.004 . 14585537

[17] Yan Y, Mumby MC. Distinct roles for PP1 and PP2A in phosphorylation of the retinoblastoma protein. PP2A regulates the activities of G_1_ cyclin-dependent kinases. J Biol Chem. 1999;274(45):31917–24. Epub 1999/11/05. 10.1074/jbc.274.45.31917 . 10542219

[18] Li L, Xie P, Chen J. In vivo studies on toxin accumulation in liver and ultrastructural changes of hepatocytes of the phytoplanktivorous bighead carp i.p.-injected with extracted microcystins. Toxicon. 2005;46(5):533–45. Epub 2005/08/09. 10.1016/j.toxicon.2005.06.025 . 16084552

[19] Jochimsen EM, Carmichael WW, An JS, Cardo DM, Cookson ST, Holmes CE, et al Liver failure and death after exposure to microcystins at a hemodialysis center in Brazil. N Engl J Med. 1998;338(13):873–8. 10.1056/NEJM199803263381304 . 9516222

[20] Chen L, Chen J, Zhang X, Xie P. A review of reproductive toxicity of microcystins. J Hazard Mater. 2016;301:381–99. 10.1016/j.jhazmat.2015.08.041 . 26521084

[21] Liu W, Chen C, Chen L, Wang L, Li J, Chen Y, et al Sex-dependent effects of microcystin-LR on hypothalamic-pituitary-gonad axis and gametogenesis of adult zebrafish. Sci Rep. 2016;6:22819 Epub 2016/03/11. 10.1038/srep22819 26960901 PMC4785373

[22] Oziol L, Bouaicha N. First evidence of estrogenic potential of the cyanobacterial heptotoxins the nodularin-R and the microcystin-LR in cultured mammalian cells. J Hazard Mater. 2010;174(1–3):610–5. Epub 2009/10/16. 10.1016/j.jhazmat.2009.09.095 . 19828236

[23] Rogers ED, Henry TB, Twiner MJ, Gouffon JS, McPherson JT, Boyer GL, et al Global gene expression profiling in larval zebrafish exposed to microcystin-LR and microcystis reveals endocrine disrupting effects of cyanobacteria. Environ Sci Technol. 2011;45(5):1962–9. Epub 2011/02/02. 10.1021/es103538b . 21280650

[24] Zhao Y, Xie L, Yan Y. Microcystin-LR impairs zebrafish reproduction by affecting oogenesis and endocrine system. Chemosphere. 2015;120:115–22. Epub 2014/07/12. 10.1016/j.chemosphere.2014.06.028 . 25014902

[25] International Programme on Chemical Safety (IPCS). Global assessment of the state-of-the-science of endocrine disruptors Geneva, Switzerland 2002.

[26] Stepankova T, Ambrozova L, Blaha L, Giesy JP, Hilscherova K. *In vitro* modulation of intracellular receptor signaling and cytotoxicity induced by extracts of cyanobacteria, complex water blooms and their fractions. Aquat Toxicol. 2011;105(3–4):497–507. Epub 2011/09/10. 10.1016/j.aquatox.2011.08.002 . 21903046

[27] Sychrova E, Stepankova T, Novakova K, Blaha L, Giesy JP, Hilscherova K. Estrogenic activity in extracts and exudates of cyanobacteria and green algae. Environ Int. 2012;39(1):134–40. Epub 2012/01/03. 10.1016/j.envint.2011.10.004 . 22208753

[28] Hecker M, Newsted JL, Murphy MB, Higley EB, Jones PD, Wu R, et al Human adrenocarcinoma (H295R) cells for rapid in vitro determination of effects on steroidogenesis: hormone production. Toxicol Appl Pharmacol. 2006;217(1):114–24. Epub 2006/09/12. 10.1016/j.taap.2006.07.007 . 16962624

[29] Sanderson JT, Boerma J, Lansbergen GW, van den Berg M. Induction and inhibition of aromatase (CYP19) activity by various classes of pesticides in H295R human adrenocortical carcinoma cells. Toxicol Appl Pharmacol. 2002;182(1):44–54. Epub 2002/07/20. 10.1006/taap.2002.9420 . 12127262

[30] La Merrill MA, Vandenberg LN, Smith MT, Goodson W, Browne P, Patisaul HB, et al Consensus on the key characteristics of endocrine-disrupting chemicals as a basis for hazard identification. Nat Rev Endocrinol. 2020;16(1):45–57. 10.1038/s41574-019-0273-8 31719706 PMC6902641

[31] Hinson JP, Raven PW. Effects of endocrine-disrupting chemicals on adrenal function. Best Pract Res Clin Endocrinol Metab. 206;20(1):111–20. 10.1016/j.beem.2005.09.006 . 16522523

[32] Miller WL. Steroid hormone synthesis in mitochondria. Mol Cell Endocrinol. 2013;379(1–2):62–73. Epub 2013/05/01. 10.1016/j.mce.2013.04.014 . 23628605

[33] Payne AH, Hales DB. Overview of steroidogenic enzymes in the pathway from cholesterol to active steroid hormones. Endocr Rev. 2004;25(6):947–70. Epub 2004/12/08. 10.1210/er.2003-0030 . 15583024

[34] Stocco DM. StAR protein and the regulation of steroid hormone biosynthesis. Annu Rev Physiol. 2001;63:193–213. Epub 2001/02/22. 10.1146/annurev.physiol.63.1.193 . 11181954

[35] Miller WL, Auchus RJ. The molecular biology, biochemistry, and physiology of human steroidogenesis and its disorders. Endocr Rev. 2011;32(1):81–151. Epub 2010/11/06. 10.1210/er.2010-0013 21051590 PMC3365799

[36] Wang L, Wang X, Geng Z, Zhou Y, Chen Y, Wu J, et al Distribution of microcystin-LR to testis of male Sprague-Dawley rats. Ecotoxicology. 2013;22(10):1555–63. Epub 2013/10/24. 10.1007/s10646-013-1141-2 . 24150695

[37] (WHO) WHO. Guidelines for Drinking-Water Quality: Fourth Edition Incorporating the First Addendum, https://www.who.int/water_sanitation_health/publications/drinking-water-quality-guidelines-4-including-1st-addendum/en/ Geneva, Switzerland 2017. 28759192

[38] Wang L, Lin W, Zha Q, Guo H, Zhang D, Yang L, et al Persistent exposure to environmental levels of microcystin-LR disturbs cortisol production via hypothalamic-pituitary-interrenal (HPI) axis and subsequently liver glucose metabolism in adult male zebrafish (Danio rerio). Toxins (Basel). 2020;12(5). Epub 2020/05/02. 10.3390/toxins12050282 . 32353954 PMC7290660

[39] Hou J, Li L, Wu N, Su Y, Lin W, Li G, et al Reproduction impairment and endocrine disruption in female zebrafish after long-term exposure to MC-LR: A life cycle assessment. Environ Pollut. 2016;208(Pt B):477–85. Epub 2015/11/11. 10.1016/j.envpol.2015.10.018 . 26552529

[40] Gazdar AF, Oie HK, Shackleton CH, Chen TR, Triche TJ, Myers CE, et al Establishment and characterization of a human adrenocortical carcinoma cell line that expresses multiple pathways of steroid biosynthesis. Cancer Res. 1990;50(17):5488–96. Epub 1990/09/01. . 2386954

[41] Gracia T, Hilscherova K, Jones PD, Newsted JL, Zhang X, Hecker M, et al The H295R system for evaluation of endocrine-disrupting effects. Ecotoxicol Environ Saf. 2006;65(3):293–305. Epub 2006/08/29. 10.1016/j.ecoenv.2006.06.012 . 16935330

[42] Hecker M, Giesy JP. Novel trends in endocrine disruptor testing: the H295R Steroidogenesis Assay for identification of inducers and inhibitors of hormone production. Anal Bioanal Chem. 2008;390(1):287–91. 10.1007/s00216-007-1657-5 . 17957359

[43] Rainey WE, Bird IM, Sawetawan C, Hanley NA, McCarthy JL, McGee EA, et al Regulation of human adrenal carcinoma cell (NCI-H295) production of C19 steroids. J Clin Endocrinol Metab. 1993;77(3):731–7. Epub 1993/09/01. 10.1210/jcem.77.3.8396576 . 8396576

[44] Rainey WE, Bird IM, Mason JI. The NCI-H295 cell line: a pluripotent model for human adrenocortical studies. Mol Cell Endocrinol. 1994;100(1–2):45–50. Epub 1994/04/01. 10.1016/0303-7207(94)90277-1 . 8056157

[45] Organisation for Economic Co-operation and Development (OECD). Revised Guidance Document 150 on Standardised Test Guidelines for Evaluating Chemicals for Endocrine Disruption2018.

[46] Janssen EM. Cyanobacterial peptides beyond microcystins—a review on co-occurrence, toxicity, and challenges for risk assessment. Water Res. 2019;151:488–99. Epub 2019/01/15. 10.1016/j.watres.2018.12.048 . 30641464

[47] Dittmann E, Neilan BA, Erhard M, Von Döhren H, Börner T. Insertional mutagenesis of a peptide synthetase gene that is responsible for hepatotoxin production in the cyanobacterium *Microcystis aeruginosa* PCC 7806. Mol Microbiol. 1997;26(4):779–87. 10.1046/j.1365-2958.1997.6131982.x 9427407

[48] Stefanelli M, Vichi S, Stipa G, Funari E, Testai E, Scardala S, et al Survival, growth and toxicity of Microcystis aeruginosa PCC7806 in experimental conditions mimicking some features of the human gastro-intestinal environment. Chem-Biol Interact. 2014;215:54–61. Epub 2014/03/29. 10.1016/j.cbi.2014.03.006 . 24667652

[49] Mallia V, Ivanova L, Eriksen GS, Harper E, Connolly L, Uhlig S. Investigation of *in vitro* endocrine activities of *Microcystis* and *Planktothrix* cyanobacterial strains. Toxins (Basel). 2020;12(4). Epub 2020/04/09. 10.3390/toxins12040228 32260386 PMC7232361

[50] Portmann C, Blom JF, Gademann K, Juttner F. Aerucyclamides A and B: isolation and synthesis of toxic ribosomal heterocyclic peptides from the cyanobacterium *Microcystis aeruginosa* PCC 7806. J Nat Prod. 2008;71(7):1193–6. Epub 2008/06/19. 10.1021/np800118g . 18558743

[51] Portmann C, Blom JF, Kaiser M, Brun R, Juttner F, Gademann K. Isolation of aerucyclamides C and D and structure revision of microcyclamide 7806A: heterocyclic ribosomal peptides from *Microcystis aeruginosa* PCC 7806 and their antiparasite evaluation. J Nat Prod. 2008;71(11):1891–6. Epub 2008/11/01. 10.1021/np800409z . 18973386

[52] Tonk L, Welker M, Huisman J, Visser PM. Production of cyanopeptolins, anabaenopeptins, and microcystins by the harmful cyanobacteria *Anabaena* 90 and *Microcystis* PCC7806. Harmful Algae. 2009;8:219–24.

[53] Tillett D, Dittmann E, Erhard M, von Dohren H, Borner T, Neilan BA. Structural organization of microcystin biosynthesis in *Microcystis aeruginosa* PCC7806: an integrated peptide-polyketide synthetase system. Chem Biol. 2000;7(10):753–64. Epub 2000/10/18. 10.1016/s1074-5521(00)00021-1 . 11033079

[54] Briand E, Bormans M, Gugger M, Dorrestein PC, Gerwick WH. Changes in secondary metabolic profiles of *Microcystis aeruginosa* strains in response to intraspecific interactions. Environ Microbiol. 2016;18(2):384–400. Epub 2015/05/20. 10.1111/1462-2920.12904 25980449 PMC5083810

[55] Mallia V, Uhlig S, Rafuse C, Meija J, Miles CO. Novel microcystins from Planktothrix prolifica NIVA-CYA 544 Identified by LC–MS/MS, functional group derivatization and 15N-labeling. Mar Drugs. 2019;17(11). Epub 2019/11/17. 10.3390/md17110643 31731697 PMC6891653

[56] Kotai J. Instructions for preparation of modified nutrient solution Z8 for algae. Norwegian Institute for Water Research, Oslo, Norway, 1972 Contract No.: Publication B-11/69).

[57] Mallia V, Uhlig S, Rafuse C, Meija J, Miles CO. Novel microcystins from Planktothrix prolifica NIVA-CYA 544 identified by LC-MS/MS, functional group derivatization and 15N-labeling. Mar Drugs. 2019;17(11). Epub 2019/11/17. 10.3390/md17110643 31731697 PMC6891653

[58] O'Brien J, Wilson I, Orton T, Pognan F. Investigation of the Alamar Blue (resazurin) fluorescent dye for the assessment of mammalian cell cytotoxicity. Eur J Biochem. 2000;267(17):5421–6. Epub 2000/08/22. 10.1046/j.1432-1327.2000.01606.x . 10951200

[59] Weisser JJ, Hansen CH, Poulsen R, Larsen LW, Cornett C, Styrishave B. Two simple cleanup methods combined with LC–MS/MS for quantification of steroid hormones in *in vivo* and *in vitro* assays. Anal Bioanal Chem. 2016;408(18):4883–95. Epub 2016/05/07. 10.1007/s00216-016-9575-z . 27150205

[60] (EMA) EMA. ICH Q2 (R1) Validation of analytical procedures: text and methodology, https://www.ema.europa.eu/en/ich-q2-r1-validation-analytical-procedures-text-methodology.

[61] Bradley W, Robert P. Multivariate analysis in metabolomics. Curr Metabolomics. 2013;1(1):92–107. 10.2174/2213235X11301010092. 26078916 PMC4465187

[62] Blasco H, Błaszczyński J, Billaut JC, Nadal-Desbarats L, Pradat PF, Devos D, et al Comparative analysis of targeted metabolomics: dominance-based rough set approach versus orthogonal partial least square-discriminant analysis. J Biomed Inf. 2015;53:291–9. Epub 2014/12/17. 10.1016/j.jbi.2014.12.001 . 25499899

[63] Lee JW, Ji SH, Choi BR, Choi DJ, Lee YG, Kim HG, et al UPLC-QTOF/MS-based metabolomics applied for the quality evaluation of four processed panax ginseng products. Molecules. 2018;23(8). Epub 2018/08/22. 10.3390/molecules23082062 30126124 PMC6222836

[64] Kellogg JJ, Todd DA, Egan JM, Raja HA, Oberlies NH, Kvalheim OM, et al Biochemometrics for natural products research: comparison of data analysis approaches and application to identification of bioactive compounds. J Nat Prod. 2016;79(2):376–86. 10.1021/acs.jnatprod.5b01014 26841051 PMC5135737

[65] Ahmed KEM, Froysa HG, Karlsen OA, Sagen JV, Mellgren G, Verhaegen S, et al LC–MS/MS based profiling and dynamic modelling of the steroidogenesis pathway in adrenocarcinoma H295R cells. Toxicol In Vitro. 2018;52:332–41. Epub 2018/07/19. 10.1016/j.tiv.2018.07.002 . 30017865

[66] Lee TC, Miller WL, Auchus RJ. Medroxyprogesterone acetate and dexamethasone are competitive inhibitors of different human steroidogenic enzymes. J Clin Endocrinol Metab. 1999;84(6):2104–10. Epub 1999/06/18. 10.1210/jcem.84.6.5646 . 10372718

[67] Jia X, Liu Z, Lu X., Tang J., Wu Y., Du Q., et al Effects of MCLR exposure on sex hormone synthesis and reproduction-related genes expression of testis in male Rana nigromaculata. Environ Poll. 2018;236;12–20. 10.1016/j.envpol.2018.01.057 . 29414332

[68] Xu Y, Xing Y, Chen Y, Chao Y, Lin Z, Fan E, et al Structure of the protein phosphatase 2A holoenzyme. Cell. 2006;127(6):1239–51. 10.1016/j.cell.2006.11.033 17174897

[69] Niedermeyer THJ, Daily A, Swiatecka-Hagenbruch M, Moscow JA. Selectivity and potency of microcystin congeners against OATP1B1 and OATP1B3 expressing cancer cells. PLOS ONE. 2014;9(3): e91476 10.1371/journal.pone.0091476 24614281 PMC3948918

[70] Asif AR, Steffgen J, Metten M, Grunewald RW, Müller GA, Bahn A, et al Presence of organic anion transporters 3 (OAT3) and 4 (OAT4) in human adrenocortical cells. Pflugers Arch–Eur J Physiol. 2005;450:88–95. 10.1007/s00424-004-1373-3 15864504

