# Supplementary material for: Microcystins and Microcystis aeruginosa PCC7806 extracts modulate steroidogenesis differentially in the human H295R adrenal model
Source: PLoS One. 2020 Dec 15;15(12):e0244000. doi: 10.1371/journal.pone.0244000 (PMC7737990; doi:10.1371/journal.pone.0244000)
Supplement: S1 Fig — The purpose of the figure is to show that hormone concentrations of the solvent and medium control samples were homogeneous across experiments thus confirming the absence of technical bias. (DOCX) [file pone.0244000.s001.docx]

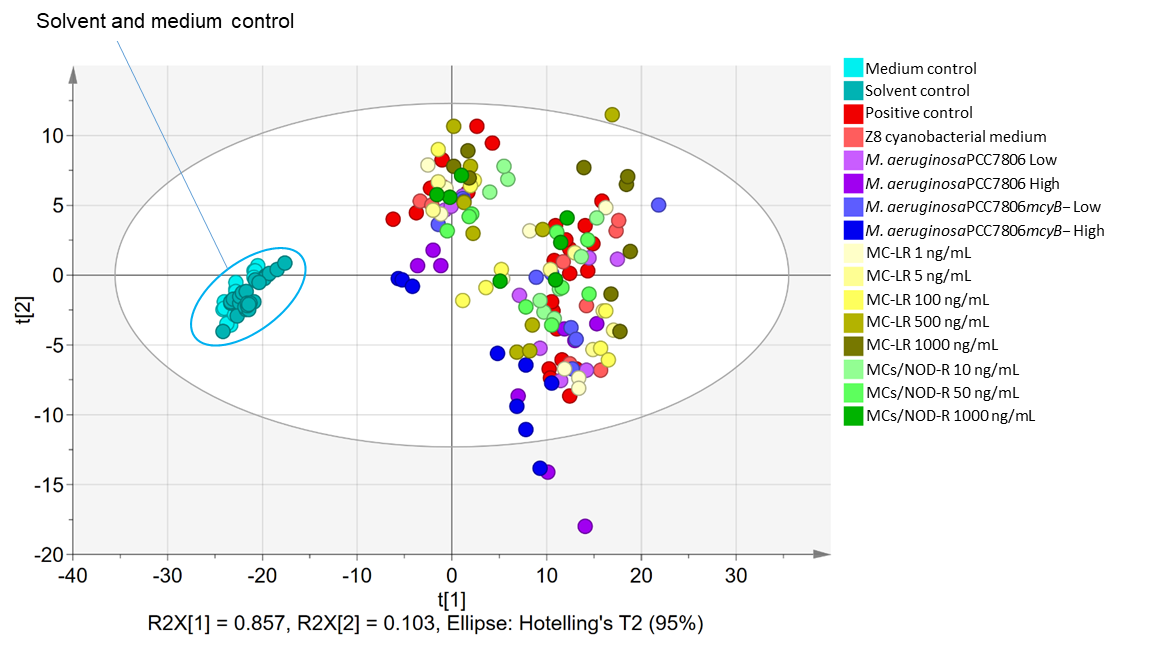


**Fig. S1.** Scores plot from principal component analysis of the pareto-scaled and log-transformed H295R hormone data. The purpose of the figure is to show that hormone concentrations of the solvent and medium control samples were homogeneous across experiments thus confirming the absence of technical bias.
