# Supplementary material for: Microcystins and Microcystis aeruginosa PCC7806 extracts modulate steroidogenesis differentially in the human H295R adrenal model
Source: PLoS One. 2020 Dec 15;15(12):e0244000. doi: 10.1371/journal.pone.0244000 (PMC7737990; doi:10.1371/journal.pone.0244000)
Supplement: S1 Scheme — This scheme was repeated in three independent runs (Expt1, Expt2, Expt3). Concentrations reported are concentration of samples in the assay (once diluted in the growth medium). (DOCX) [file pone.0244000.s004.docx]

| MEDIUM  CONTROL | MEDIUM  CONTROL | MEDIUM  CONTROL | SOLVENT  CONTROL | SOLVENT  CONTROL | SOLVENT  CONTROL |
| --- | --- | --- | --- | --- | --- |
| POSITIVE  CONTROL | POSITIVE CONTROL | POSITIVE CONTROL | Z8  MEDIUM | Z8  MEDIUM | Z8  MEDIUM |
| PCC7806  low (5ng/ml) | PCC7806  low (5ng/ml) | PCC7806  low (5ng/ml) | PCC7806  high (500ng/ml) | PCC7806  high (500ng/ml | PCC7806  high (500ng/ml |
| PCC7806*mcyB*- low conc. | PCC7806*mcyB*- low conc. | PCC7806*mcyB*- low conc. | PCC7806*mcyB*- high conc. | PCC7806*mcyB*- high conc. | PCC7806*mcyB*- high conc. |
| PLATE 1 |  |  |  |  |  |
| MEDIUM  CONTROL | MEDIUM  CONTROL | MEDIUM  CONTROL | SOLVENT  CONTROL | SOLVENT  CONTROL | SOLVENT  CONTROL |
| POSITIVE  CONTROL | POSITIVE CONTROL | POSITIVE CONTROL | MC-LR 1ng/ml | MC-LR 1ng/ml | MC-LR 1ng/ml |
| MC-LR 5ng/ml | MC-LR 5ng/ml | MC-LR 5ng/ml | MC-LR 100ng/ml | MC-LR 100ng/ml | MC-LR 100ng/ml |
| MC-LR 500ng/ml | MC-LR 500ng/ml | MC-LR 500ng/ml | MC-LR 1000ng/ml | MC-LR 1000ng/ml | MC-LR 1000ng/ml |
| PLATE 2 |  |  |  |  |  |
| MEDIUM  CONTROL | MEDIUM  CONTROL | MEDIUM  CONTROL | SOLVENT  CONTROL | SOLVENT  CONTROL | SOLVENT  CONTROL |
| POSITIVE  CONTROL | POSITIVE CONTROL | POSITIVE CONTROL | mix MCs/NOD-R 1ng/ml | mix MCs/NOD-R 1ng/ml | mix MCs/NOD-R 1ng/ml |
| mix MCs/NOD-R 5ng/ml | mix MCs/NOD-R 5ng/ml | mix MCs/NOD-R 5ng/ml | mix MCs/NOD-R 100ng/ml | *mix MCs/NOD-R 100ng/ml | *mix MCs/NOD-R 100ng/ml |
|  |  |  |  |  |  |
| PLATE 3 |  |  |  |  |  |

MEDIUM CONTROL = 100% complete growth medium (D-MEM/F-12 (1x) liquid 1:1 +1% ITS +5% FBS)

SOLVENT CONTROL = growth medium with 0.5% methanol

POSITIVE CONTROL = growth medium with 0.5% methanol and 1.5uM forskolin stimulation

MC-LR = microcystin-LR

mix MCs/NOD-R = standard mix of 9 microcystins including MC-LR + nodularin-R

All exposure samples were prepared as 990uL growth medium + 10uL specific mother solution.

* not included in the 3^rd^ plate of the 3^rd^ run

**Scheme 1.** Experimental plan for H295R exposure in three 24-well plates. This scheme was repeated in three independent runs (Expt1, Expt2, Expt3). Concentrations reported are concentration of samples in the assay (once diluted in the growth medium).
